# Crystal structure of *N*-[4-amino-5-cyano-6-(methyl­sulfan­yl)pyridin-2-yl]acetamide hemihydrate

**DOI:** 10.1107/S205698901500256X

**Published:** 2015-02-13

**Authors:** Mehmet Akkurt, Kyle S. Knight, Shaaban K. Mohamed, Bahgat R. M. Hussein, Mustafa R. Albayati

**Affiliations:** a Department of Physics, Faculty of Sciences, Erciyes University, 38039 Kayseri, Turkey; b Department of Chemistry, The University of Tennessee at Chattanooga, Chattanooga, TN 37403, USA; c Chemistry and Environmental Division, Manchester Metropolitan University, Manchester M1 5GD, England; d Chemistry Department, Faculty of Science, Minia University, 61519 El-Minia, Egypt; e Chemistry Department, Faculty of Science, Sohag University, 82524 Sohag, Egypt; f Kirkuk University, College of Science, Department of Chemistry, Kirkuk, Iraq

**Keywords:** crystal structure, poly-functional pyridines, acetamide, disorder, hydrogen bonding

## Abstract

The title compound, C_9_H_10_N_4_OS·0.5H_2_O, crystallizes with two independent mol­ecules (*A* and *B*) in the asymmetric unit, together with a water mol­ecule of crystallization. The acetamide moiety, which has an extended conformation, is inclined to the pyridine ring by 7.95 (16)° in mol­ecule *A* and by 1.77 (16)° in mol­ecule *B*. In the crystal, the *A* and *B* mol­ecules are linked by two N—H⋯O_carbon­yl_ hydrogen bonds, forming a dimer. The dimers are linked *via* N—H⋯N hydrogen bonds, forming ribbons that are linked by N—H⋯O_water_ hydrogen bonds to form sheets parallel to (110). The sheets are linked by O—H⋯N hydrogen bonds, forming slabs, and between the slabs there are weak slipped parallel π–π inter­actions [inter-centroid distance = 3.734 (2) Å, inter­planar distance = 3.3505 (11) Å and slippage = 1.648 Å], forming a three-dimensional structure.

## Related literature   

For various applications of polyfunctional pyridines, see: Knyazhanskii *et al.* (1996 ▸); Kurfurst *et al.* (1989 ▸); Enyedy *et al.* (2003 ▸); Arora & Knaus (1999 ▸); Kim *et al.* 2004 ▸); Pillai *et al.* (2003 ▸).
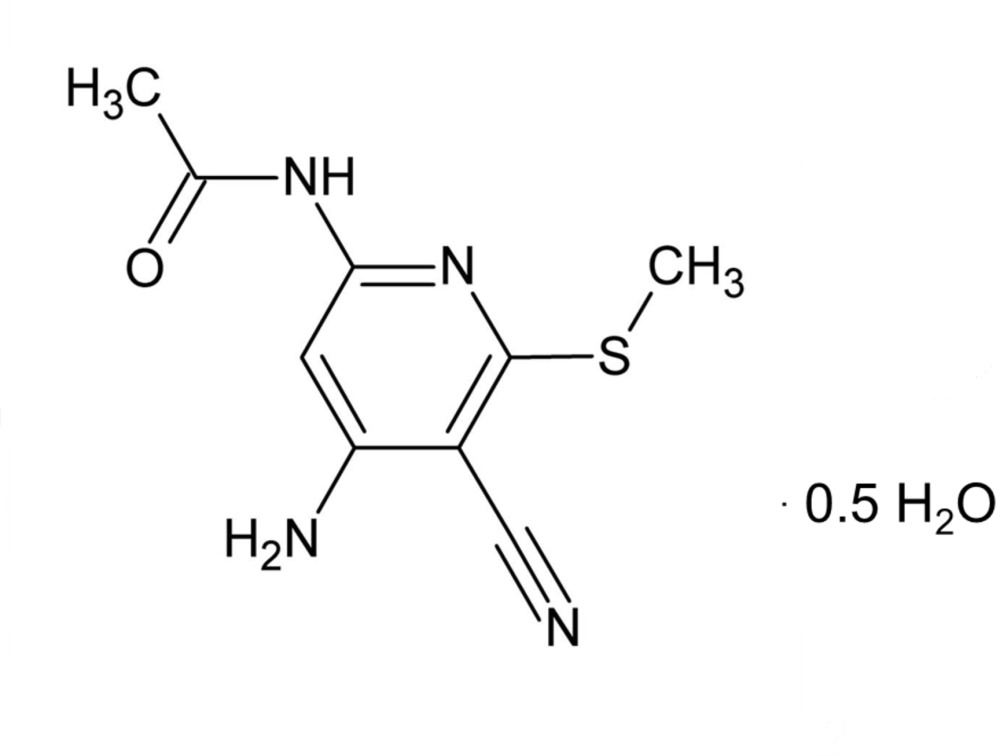


## Experimental   

### Crystal data  

C_9_H_10_N_4_OS·0.5H_2_O
*M* *_r_* = 231.28
Triclinic, 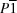

*a* = 8.229 (3) Å
*b* = 10.181 (4) Å
*c* = 13.198 (5) Å
α = 84.221 (10)°
β = 80.036 (10)°
γ = 82.136 (11)°
*V* = 1075.4 (7) Å^3^
*Z* = 4
Mo *K*α radiation
μ = 0.29 mm^−1^
*T* = 200 K
0.40 × 0.40 × 0.40 mm

### Data collection  

Bruker SMART X2S benchtop diffractometer
Absorption correction: multi-scan (*SADABS*; Bruker, 2009 ▸) *T*_min_ = 0.813, *T*_max_ = 1.000
19554 measured reflections
3775 independent reflections
2517 reflections with *I* > 2σ(*I*)
*R*_int_ = 0.066

### Refinement  

*R*[*F* ^2^ > 2σ(*F* ^2^)] = 0.052
*wR*(*F* ^2^) = 0.150
*S* = 1.03
3775 reflections
305 parameters
9 restraints
H atoms treated by a mixture of independent and constrained refinement
Δρ_max_ = 0.63 e Å^−3^
Δρ_min_ = −0.31 e Å^−3^

### 

Data collection: *APEX2* (Bruker, 2009 ▸); cell refinement: *SAINT* (Bruker, 2009 ▸); data reduction: *SAINT*; program(s) used to solve structure: *SHELXS2014* (Sheldrick, 2008 ▸); program(s) used to refine structure: *SHELXL2014* (Sheldrick, 2015 ▸); molecular graphics: *PLATON* (Spek, 2009 ▸); software used to prepare material for publication: *SHELXL2014* and *PLATON*.

## Supplementary Material

Crystal structure: contains datablock(s) global, I. DOI: 10.1107/S205698901500256X/su5077sup1.cif

Structure factors: contains datablock(s) I. DOI: 10.1107/S205698901500256X/su5077Isup2.hkl

Supporting information file. DOI: 10.1107/S205698901500256X/su5077Isup3.cml

**Fig. 1.**
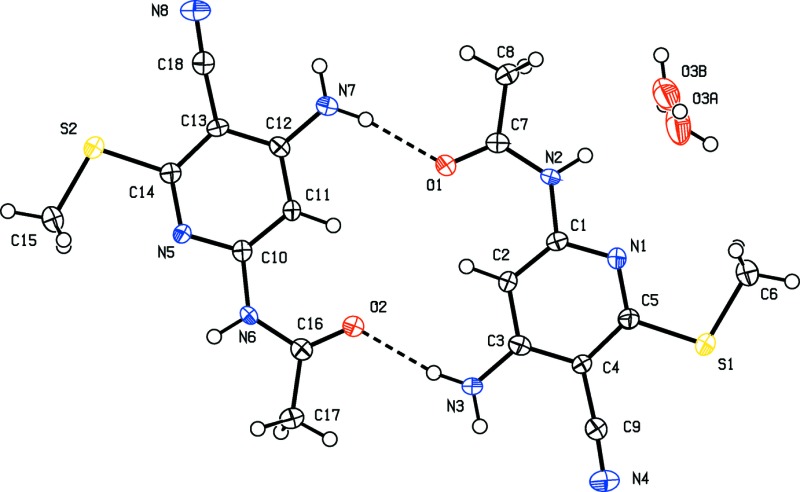
Molecular structure of the title compound, with atom labelling. Displacement ellipsoids are drawn at the 50% probability level. The N—H···O hydrogen bonds are shown as dashed lines (see Table 1 for details).

**Fig. 2.**
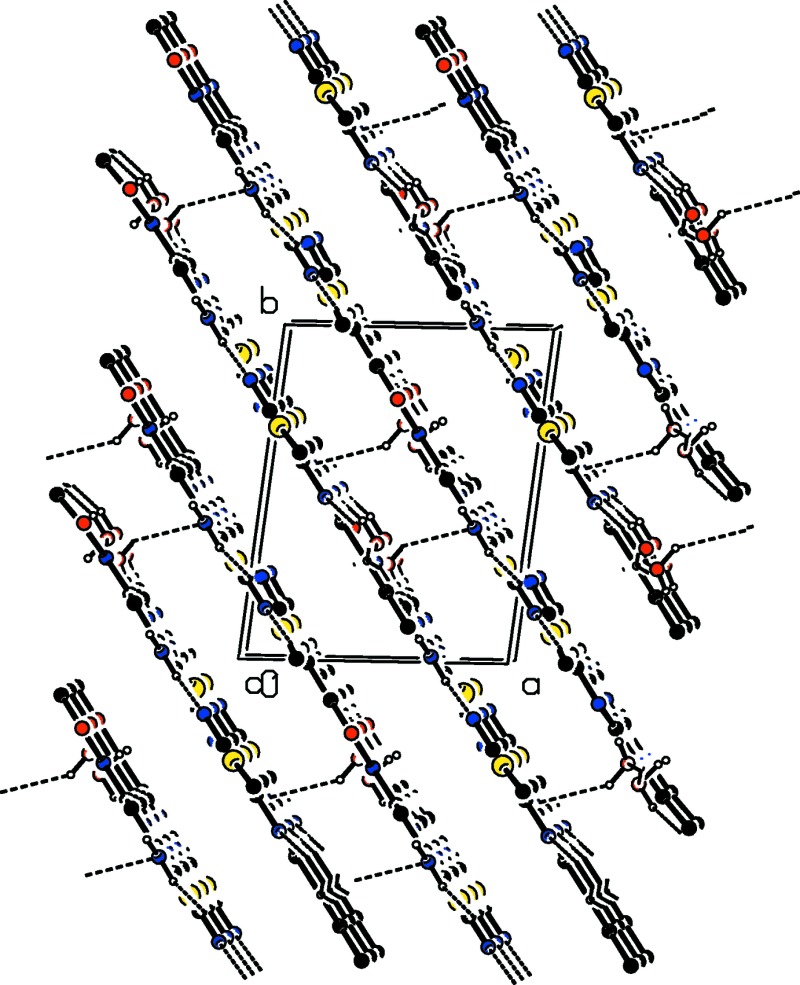
View along the c axis of the crystal packing of the title compound. The hydrogen bonds are shown as dashed lines (see Table 1 for details; H atoms not involved in hydrogen bonding have been omitted for clarity).

. DOI: 10.1107/S205698901500256X/su5077fig1.tif
Mol­ecular structure of the title compound, with atom labelling. Displacement ellipsoids are drawn at the 50% probability level. The N—H⋯O hydrogen bonds are shown as dashed lines (see Table 1 for details).

c . DOI: 10.1107/S205698901500256X/su5077fig2.tif
View along the *c* axis of the crystal packing of the title compound. The hydrogen bonds are shown as dashed lines (see Table 1 for details; H atoms not involved in hydrogen bonding have been omitted for clarity).

CCDC reference: 1048267

Additional supporting information:  crystallographic information; 3D view; checkCIF report

## Figures and Tables

**Table 1:** Hydrogen-bond geometry (, )

*D*H*A*	*D*H	H*A*	*D* *A*	*D*H*A*
N3H3*AN*O2	0.88	2.00	2.870(3)	169
N7H7*BN*O1	0.88	1.99	2.860(3)	170
N3H3*BN*N8^i^	0.88	2.33	3.080(4)	143
N7H7*AN*N4^ii^	0.88	2.38	3.116(4)	142
N2H2*N*O3*A*	0.88	2.12	2.984(10)	166
N2H2*N*O3*B*	0.88	2.21	3.079(9)	172
N6H6*N*O3*A* ^iii^	0.88	2.26	3.138(10)	173
N6H6*N*O3*B* ^iii^	0.88	2.19	3.051(9)	166
O3*A*H3*A*2N7^iv^	0.85(2)	2.36(6)	3.083(12)	143(9)
O3*B*H3*B*1N5^v^	0.85(2)	2.57(6)	3.239(8)	137(8)

Symmetry codes: (i) 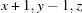; (ii) 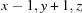; (iii) 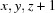; (iv) 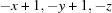; (v) 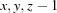.

## supplementary crystallographic information

### S1. Comment

Poly-functional pyridines are an interesting class of compounds due to their optical properties (Knyazhanskii *et al.*, 1996; Kurfurst *et al.*, 1989), and their biological activities (Enyedy *et al.*, 2003), such as anticonvulsants (Arora *et al.*, 1999), antihistaminic reagents (Kim *et al.*, 2004), and cardivascular disorder treatments (Pillai *et al.*, 2003). In view of such facts we herein report on the synthesis and crystal structure of the new title poly-functional pyridine compound.

The asymmetric unit of the title compound, Fig. 1, contains two independent title molecules (A and B) and one water molecule. In molecule A the dihedral angle between the pyridine ring (N1/C1–C5) and the acetamide moiety (O1/N2(C7/C8) is 7.95 (16)°, while in molecule B the corresponding angle, involving the pyridine ring (N5/C10-C14) and actemide moiety (O2/N2/C7/C8), is 1.77 (16)°.

In the crystal, the A and B molecules are linked by two N-H···O_carbonyl_ hydrogen bonds forming a dimer (Table 1 and Fig. 1). The dimers are linked via N-H···N hydrogen bonds forming ribbons that are linked by N-H···O_water_ hydrogen bonds to form sheets parallel to (110); see Table 1. The sheets are linked by O-H···N hydrogen bonds forming slabs (Table 1). Between the slabs there are weak slipped parallel π-π interactions forming a three-dimensional structure, Fig. 2 [inter-centroid distance Cg1···Cg1^i^ = 3.734 (2) Å, inter-planar distance = 3.351 (1) Å, slippage = 1.648 Å; Cg1 is the centroid of ring N5/C10-C14 (molecule B); symmetry code: (i) -x, -y+1, -z+1].

### S2. Experimental

A solution of 0.5 g (2.7 mmol) of 4,6-diamino-3-cyano-2-methylthiopyridine-2(1*H*)-thione in 30 ml glacial acetic acid was refluxed for 3 h. The reaction mixture was allowed to cool and it was then poured into 100 ml of ice cold water. The formed precipitate was collected and dried under vacuum. Yellow crystals, suitable for X-ray analysis, were obtained by recrystallization of the solid product from ethanol (yield: 92%; m.p.: 523–525 K).

### S3. Refinement

The water molecule O3 is disordered over two positions (O3A/O3B) and was refined with an occupancy ratio of 0.5:0.5. The H atoms were included in calculated positions (calc-OH in WinGX; Farrugia, 2012 and refined with distance restraints: O—H = 0.84 (2) Å and H···H = 1.35 (2) Å with U_iso_(H) = 1.5U_eq_(O). The C and N-bound H atoms were placed in calculated positions and treated as riding atoms: C—H = 0.95 - 0.98 Å and N—H = 0.88 Å, with U_iso_(H) = 1.5U_eq_(C) for methyl H atoms and = 1.2U_eq_(N,C) for other H atoms.

### Crystal data

**Table:**

C_9_H_10_N_4_OS·0.5H_2_O	*Z* = 4
*M**_r_* = 231.28	*F*(000) = 484
Triclinic, *P*1	*D*_x_ = 1.428 Mg m^−^^3^
*a* = 8.229 (3) Å	Mo *K*α radiation, λ = 0.71073 Å
*b* = 10.181 (4) Å	Cell parameters from 4579 reflections
*c* = 13.198 (5) Å	θ = 2.5–25.1°
α = 84.221 (10)°	µ = 0.29 mm^−^^1^
β = 80.036 (10)°	*T* = 200 K
γ = 82.136 (11)°	Block, yellow
*V* = 1075.4 (7) Å^3^	0.40 × 0.40 × 0.40 mm

### Data collection

**Table:**

Bruker SMART X2S benchtop diffractometer	3775 independent reflections
Radiation source: XOS X-beam microfocus source	2517 reflections with *I* > 2σ(*I*)
Doubly curved silicon crystal monochromator	*R*_int_ = 0.066
ω scans	θ_max_ = 25.2°, θ_min_ = 2.5°
Absorption correction: multi-scan (*SADABS*; Bruker, 2009)	*h* = −9→9
*T*_min_ = 0.813, *T*_max_ = 1.000	*k* = −12→12
19554 measured reflections	*l* = −15→15

### Refinement

**Table:**

Refinement on *F*^2^	Primary atom site location: structure-invariant direct methods
Least-squares matrix: full	Secondary atom site location: difference Fourier map
*R*[*F*^2^ > 2σ(*F*^2^)] = 0.052	Hydrogen site location: mixed
*wR*(*F*^2^) = 0.150	H atoms treated by a mixture of independent and constrained refinement
*S* = 1.03	*w* = 1/[σ^2^(*F*_o_^2^) + (0.0859*P*)^2^ + 0.1759*P*] where *P* = (*F*_o_^2^ + 2*F*_c_^2^)/3
3775 reflections	(Δ/σ)_max_ = 0.001
305 parameters	Δρ_max_ = 0.63 e Å^−^^3^
9 restraints	Δρ_min_ = −0.31 e Å^−^^3^

### Special details

**Table:**

Geometry. All esds (except the esd in the dihedral angle between two l.s. planes) are estimated using the full covariance matrix. The cell esds are taken into account individually in the estimation of esds in distances, angles and torsion angles; correlations between esds in cell parameters are only used when they are defined by crystal symmetry. An approximate (isotropic) treatment of cell esds is used for estimating esds involving l.s. planes.

### Fractional atomic coordinates and isotropic or equivalent isotropic displacement parameters (Å^2^)

**Table:**

	*x*	*y*	*z*	*U*_iso_*/*U*_eq_	Occ. (<1)
S1	0.84898 (9)	−0.09268 (8)	−0.11814 (5)	0.0324 (2)	
O1	0.3621 (3)	0.3985 (2)	0.14350 (16)	0.0444 (6)	
N1	0.6422 (3)	0.1137 (2)	−0.04595 (17)	0.0243 (5)	
N2	0.4586 (3)	0.2943 (2)	−0.00302 (17)	0.0263 (6)	
H2N	0.4539	0.3015	−0.0695	0.032*	
N3	0.7191 (3)	0.0201 (3)	0.25778 (18)	0.0369 (7)	
H3AN	0.6676	0.0723	0.3056	0.044*	
H3BN	0.7858	−0.0510	0.2744	0.044*	
N4	0.9584 (4)	−0.2438 (3)	0.1290 (2)	0.0453 (7)	
C1	0.5671 (3)	0.1876 (3)	0.0309 (2)	0.0234 (6)	
C2	0.5899 (3)	0.1621 (3)	0.1320 (2)	0.0267 (7)	
H2	0.5348	0.2200	0.1827	0.032*	
C3	0.6957 (3)	0.0492 (3)	0.1588 (2)	0.0263 (7)	
C4	0.7727 (3)	−0.0312 (3)	0.0799 (2)	0.0224 (6)	
C5	0.7434 (3)	0.0062 (3)	−0.0213 (2)	0.0229 (6)	
C6	0.7793 (4)	−0.0068 (3)	−0.2309 (2)	0.0440 (9)	
H6A	0.8123	0.0831	−0.2403	0.066*	
H6B	0.6579	−0.0013	−0.2227	0.066*	
H6C	0.8295	−0.0553	−0.2914	0.066*	
C7	0.3596 (3)	0.3882 (3)	0.0525 (2)	0.0286 (7)	
C8	0.2446 (4)	0.4802 (3)	−0.0064 (2)	0.0359 (8)	
H8A	0.1873	0.5507	0.0368	0.054*	
H8B	0.1626	0.4302	−0.0258	0.054*	
H8C	0.3088	0.5199	−0.0688	0.054*	
C9	0.8785 (4)	−0.1495 (3)	0.1042 (2)	0.0290 (7)	
S2	0.05057 (10)	0.70420 (8)	0.65341 (6)	0.0356 (3)	
O2	0.5272 (3)	0.2080 (2)	0.39329 (15)	0.0378 (5)	
N5	0.2537 (3)	0.4955 (2)	0.58226 (16)	0.0215 (5)	
N6	0.4339 (3)	0.3125 (2)	0.53944 (17)	0.0255 (5)	
H6N	0.4420	0.3077	0.6053	0.031*	
N7	0.1738 (3)	0.5867 (3)	0.27873 (18)	0.0362 (7)	
H7AN	0.1079	0.6583	0.2620	0.043*	
H7BN	0.2260	0.5349	0.2308	0.043*	
N8	−0.0881 (3)	0.8386 (3)	0.4093 (2)	0.0424 (7)	
C10	0.3278 (3)	0.4203 (3)	0.5052 (2)	0.0236 (6)	
C11	0.3036 (3)	0.4451 (3)	0.4037 (2)	0.0256 (6)	
H11	0.3597	0.3881	0.3526	0.031*	
C12	0.1947 (3)	0.5562 (3)	0.3782 (2)	0.0240 (6)	
C13	0.1145 (3)	0.6354 (3)	0.4567 (2)	0.0251 (7)	
C14	0.1495 (3)	0.6013 (3)	0.5577 (2)	0.0251 (6)	
C15	0.1416 (4)	0.6296 (3)	0.7623 (2)	0.0427 (9)	
H15C	0.0914	0.6770	0.8233	0.064*	
H15B	0.2615	0.6348	0.7486	0.064*	
H15A	0.1216	0.5362	0.7747	0.064*	
C16	0.5260 (3)	0.2144 (3)	0.4854 (2)	0.0262 (7)	
C17	0.6252 (4)	0.1123 (3)	0.5476 (2)	0.0337 (7)	
H17A	0.5529	0.0487	0.5854	0.051*	
H17B	0.6719	0.1564	0.5967	0.051*	
H17C	0.7157	0.0652	0.5014	0.051*	
C18	0.0001 (4)	0.7485 (3)	0.4338 (2)	0.0298 (7)	
O3A	0.4983 (13)	0.2878 (10)	−0.2316 (7)	0.066 (3)	0.5
H3A1	0.541 (6)	0.223 (3)	−0.2637 (16)	0.099*	0.5
H3A2	0.555 (10)	0.352 (5)	−0.254 (6)	0.099*	0.5
O3B	0.4470 (11)	0.3490 (9)	−0.2356 (7)	0.052 (2)	0.5
H3B1	0.383 (8)	0.416 (5)	−0.255 (7)	0.077*	0.5
H3B2	0.386 (8)	0.289 (6)	−0.214 (2)	0.077*	0.5

### Atomic displacement parameters (Å^2^)

**Table:**

	*U*^11^	*U*^22^	*U*^33^	*U*^12^	*U*^13^	*U*^23^
S1	0.0361 (5)	0.0342 (5)	0.0234 (4)	0.0139 (4)	−0.0053 (3)	−0.0092 (3)
O1	0.0625 (15)	0.0445 (15)	0.0215 (12)	0.0279 (12)	−0.0163 (10)	−0.0105 (10)
N1	0.0268 (12)	0.0269 (14)	0.0176 (12)	0.0047 (11)	−0.0036 (10)	−0.0051 (10)
N2	0.0342 (13)	0.0255 (14)	0.0165 (12)	0.0124 (11)	−0.0094 (10)	−0.0014 (10)
N3	0.0503 (16)	0.0343 (16)	0.0206 (13)	0.0244 (13)	−0.0121 (11)	−0.0046 (11)
N4	0.0523 (17)	0.0385 (18)	0.0426 (17)	0.0174 (15)	−0.0188 (14)	−0.0031 (14)
C1	0.0246 (14)	0.0214 (15)	0.0222 (14)	0.0048 (12)	−0.0043 (11)	−0.0014 (12)
C2	0.0325 (15)	0.0264 (17)	0.0194 (14)	0.0093 (13)	−0.0059 (12)	−0.0069 (12)
C3	0.0306 (15)	0.0257 (17)	0.0226 (15)	0.0035 (13)	−0.0098 (12)	−0.0014 (12)
C4	0.0235 (14)	0.0223 (15)	0.0199 (14)	0.0059 (12)	−0.0051 (11)	−0.0040 (11)
C5	0.0217 (14)	0.0245 (16)	0.0226 (15)	0.0017 (12)	−0.0060 (11)	−0.0042 (12)
C6	0.054 (2)	0.049 (2)	0.0224 (16)	0.0151 (17)	−0.0047 (15)	−0.0050 (15)
C7	0.0321 (16)	0.0274 (17)	0.0244 (16)	0.0072 (13)	−0.0079 (13)	−0.0014 (13)
C8	0.0411 (18)	0.0340 (19)	0.0298 (17)	0.0164 (15)	−0.0111 (14)	−0.0085 (14)
C9	0.0347 (16)	0.0286 (18)	0.0233 (15)	0.0044 (15)	−0.0085 (13)	−0.0046 (13)
S2	0.0450 (5)	0.0339 (5)	0.0249 (4)	0.0171 (4)	−0.0098 (3)	−0.0110 (3)
O2	0.0507 (13)	0.0362 (13)	0.0234 (11)	0.0194 (10)	−0.0134 (10)	−0.0081 (9)
N5	0.0245 (12)	0.0211 (13)	0.0185 (12)	0.0055 (10)	−0.0059 (9)	−0.0061 (10)
N6	0.0319 (13)	0.0292 (14)	0.0139 (11)	0.0102 (11)	−0.0090 (10)	−0.0042 (10)
N7	0.0516 (16)	0.0314 (15)	0.0213 (13)	0.0178 (13)	−0.0114 (12)	−0.0028 (11)
N8	0.0465 (16)	0.0372 (17)	0.0392 (16)	0.0147 (14)	−0.0137 (13)	0.0030 (13)
C10	0.0263 (15)	0.0249 (16)	0.0189 (14)	0.0021 (13)	−0.0042 (11)	−0.0042 (12)
C11	0.0324 (15)	0.0262 (16)	0.0174 (14)	0.0078 (13)	−0.0071 (12)	−0.0072 (12)
C12	0.0286 (15)	0.0252 (16)	0.0184 (14)	0.0013 (13)	−0.0078 (11)	−0.0016 (12)
C13	0.0284 (15)	0.0213 (16)	0.0241 (15)	0.0080 (13)	−0.0084 (12)	−0.0030 (12)
C14	0.0279 (15)	0.0246 (16)	0.0222 (15)	0.0042 (13)	−0.0072 (12)	−0.0041 (12)
C15	0.056 (2)	0.046 (2)	0.0249 (16)	0.0141 (17)	−0.0152 (15)	−0.0120 (15)
C16	0.0281 (15)	0.0284 (17)	0.0219 (15)	0.0018 (13)	−0.0066 (12)	−0.0028 (13)
C17	0.0413 (18)	0.0279 (17)	0.0300 (17)	0.0108 (14)	−0.0107 (14)	−0.0044 (13)
C18	0.0358 (17)	0.0283 (18)	0.0238 (15)	0.0029 (15)	−0.0052 (13)	−0.0039 (13)
O3A	0.077 (7)	0.086 (8)	0.031 (3)	−0.003 (5)	0.002 (4)	−0.017 (5)
O3B	0.050 (5)	0.072 (6)	0.029 (3)	0.004 (4)	−0.006 (3)	−0.005 (4)

### Geometric parameters (Å, º)

**Table:**

S1—C5	1.743 (3)	O2—C16	1.222 (3)
S1—C6	1.790 (3)	N5—C14	1.332 (3)
O1—C7	1.219 (3)	N5—C10	1.345 (3)
N1—C5	1.330 (4)	N6—C16	1.353 (3)
N1—C1	1.336 (4)	N6—C10	1.394 (3)
N2—C7	1.356 (4)	N6—H6N	0.8800
N2—C1	1.398 (3)	N7—C12	1.353 (3)
N2—H2N	0.8800	N7—H7AN	0.8800
N3—C3	1.350 (3)	N7—H7BN	0.8800
N3—H3AN	0.8800	N8—C18	1.145 (4)
N3—H3BN	0.8800	C10—C11	1.381 (4)
N4—C9	1.140 (4)	C11—C12	1.395 (4)
C1—C2	1.374 (4)	C11—H11	0.9500
C2—C3	1.399 (4)	C12—C13	1.395 (4)
C2—H2	0.9500	C13—C14	1.411 (4)
C3—C4	1.399 (4)	C13—C18	1.427 (4)
C4—C5	1.403 (4)	C15—H15C	0.9800
C4—C9	1.430 (4)	C15—H15B	0.9800
C6—H6A	0.9800	C15—H15A	0.9800
C6—H6B	0.9800	C16—C17	1.500 (4)
C6—H6C	0.9800	C17—H17A	0.9800
C7—C8	1.497 (4)	C17—H17B	0.9800
C8—H8A	0.9800	C17—H17C	0.9800
C8—H8B	0.9800	O3A—H3A1	0.823 (18)
C8—H8C	0.9800	O3A—H3A2	0.85 (2)
S2—C14	1.742 (3)	O3B—H3B1	0.85 (2)
S2—C15	1.789 (3)	O3B—H3B2	0.84 (2)
			
C5—S1—C6	102.00 (14)	C14—N5—C10	116.8 (2)
C5—N1—C1	116.9 (2)	C16—N6—C10	129.0 (2)
C7—N2—C1	128.8 (2)	C16—N6—H6N	115.5
C7—N2—H2N	115.6	C10—N6—H6N	115.5
C1—N2—H2N	115.6	C12—N7—H7AN	120.0
C3—N3—H3AN	120.0	C12—N7—H7BN	120.0
C3—N3—H3BN	120.0	H7AN—N7—H7BN	120.0
H3AN—N3—H3BN	120.0	N5—C10—C11	124.8 (3)
N1—C1—C2	124.8 (3)	N5—C10—N6	111.9 (2)
N1—C1—N2	112.0 (2)	C11—C10—N6	123.2 (2)
C2—C1—N2	123.2 (3)	C10—C11—C12	118.2 (2)
C1—C2—C3	118.7 (3)	C10—C11—H11	120.9
C1—C2—H2	120.6	C12—C11—H11	120.9
C3—C2—H2	120.6	N7—C12—C13	122.0 (3)
N3—C3—C2	120.2 (3)	N7—C12—C11	119.7 (3)
N3—C3—C4	122.4 (3)	C13—C12—C11	118.3 (2)
C2—C3—C4	117.3 (2)	C12—C13—C14	118.7 (2)
C3—C4—C5	119.1 (2)	C12—C13—C18	119.8 (2)
C3—C4—C9	119.3 (2)	C14—C13—C18	121.5 (2)
C5—C4—C9	121.6 (2)	N5—C14—C13	123.2 (2)
N1—C5—C4	123.1 (2)	N5—C14—S2	119.3 (2)
N1—C5—S1	119.3 (2)	C13—C14—S2	117.6 (2)
C4—C5—S1	117.6 (2)	S2—C15—H15C	109.5
S1—C6—H6A	109.5	S2—C15—H15B	109.5
S1—C6—H6B	109.5	H15C—C15—H15B	109.5
H6A—C6—H6B	109.5	S2—C15—H15A	109.5
S1—C6—H6C	109.5	H15C—C15—H15A	109.5
H6A—C6—H6C	109.5	H15B—C15—H15A	109.5
H6B—C6—H6C	109.5	O2—C16—N6	122.9 (2)
O1—C7—N2	123.4 (2)	O2—C16—C17	122.4 (3)
O1—C7—C8	121.8 (3)	N6—C16—C17	114.8 (2)
N2—C7—C8	114.8 (2)	C16—C17—H17A	109.5
C7—C8—H8A	109.5	C16—C17—H17B	109.5
C7—C8—H8B	109.5	H17A—C17—H17B	109.5
H8A—C8—H8B	109.5	C16—C17—H17C	109.5
C7—C8—H8C	109.5	H17A—C17—H17C	109.5
H8A—C8—H8C	109.5	H17B—C17—H17C	109.5
H8B—C8—H8C	109.5	N8—C18—C13	176.0 (3)
N4—C9—C4	176.3 (3)	H3A1—O3A—H3A2	108 (3)
C14—S2—C15	101.47 (14)	H3B1—O3B—H3B2	106 (3)
			
C5—N1—C1—C2	1.5 (4)	C14—N5—C10—C11	0.1 (4)
C5—N1—C1—N2	−177.4 (2)	C14—N5—C10—N6	−180.0 (2)
C7—N2—C1—N1	178.7 (3)	C16—N6—C10—N5	178.3 (3)
C7—N2—C1—C2	−0.2 (5)	C16—N6—C10—C11	−1.7 (4)
N1—C1—C2—C3	−1.7 (4)	N5—C10—C11—C12	0.0 (4)
N2—C1—C2—C3	177.1 (3)	N6—C10—C11—C12	−179.9 (2)
C1—C2—C3—N3	−179.1 (3)	C10—C11—C12—N7	177.1 (3)
C1—C2—C3—C4	0.1 (4)	C10—C11—C12—C13	−0.8 (4)
N3—C3—C4—C5	−179.5 (3)	N7—C12—C13—C14	−176.5 (3)
C2—C3—C4—C5	1.4 (4)	C11—C12—C13—C14	1.3 (4)
N3—C3—C4—C9	0.8 (4)	N7—C12—C13—C18	3.2 (4)
C2—C3—C4—C9	−178.4 (2)	C11—C12—C13—C18	−179.0 (3)
C1—N1—C5—C4	0.1 (4)	C10—N5—C14—C13	0.5 (4)
C1—N1—C5—S1	−178.62 (19)	C10—N5—C14—S2	−179.4 (2)
C3—C4—C5—N1	−1.6 (4)	C12—C13—C14—N5	−1.2 (4)
C9—C4—C5—N1	178.2 (3)	C18—C13—C14—N5	179.1 (3)
C3—C4—C5—S1	177.2 (2)	C12—C13—C14—S2	178.7 (2)
C9—C4—C5—S1	−3.1 (4)	C18—C13—C14—S2	−1.1 (4)
C6—S1—C5—N1	0.3 (3)	C15—S2—C14—N5	3.4 (3)
C6—S1—C5—C4	−178.5 (2)	C15—S2—C14—C13	−176.4 (2)
C1—N2—C7—O1	6.3 (5)	C10—N6—C16—O2	0.0 (5)
C1—N2—C7—C8	−173.6 (3)	C10—N6—C16—C17	−179.2 (3)

### Hydrogen-bond geometry (Å, º)

**Table:**

*D*—H···*A*	*D*—H	H···*A*	*D*···*A*	*D*—H···*A*
N3—H3*AN*···O2	0.88	2.00	2.870 (3)	169
N7—H7*BN*···O1	0.88	1.99	2.860 (3)	170
N3—H3*BN*···N8^i^	0.88	2.33	3.080 (4)	143
N7—H7*AN*···N4^ii^	0.88	2.38	3.116 (4)	142
N2—H2*N*···O3*A*	0.88	2.12	2.984 (10)	166
N2—H2*N*···O3*B*	0.88	2.21	3.079 (9)	172
N6—H6*N*···O3*A*^iii^	0.88	2.26	3.138 (10)	173
N6—H6*N*···O3*B*^iii^	0.88	2.19	3.051 (9)	166
O3*A*—H3*A*2···N7^iv^	0.85 (2)	2.36 (6)	3.083 (12)	143 (9)
O3*B*—H3*B*1···N5^v^	0.85 (2)	2.57 (6)	3.239 (8)	137 (8)

Symmetry codes: (i) *x*+1, *y*−1, *z*; (ii) *x*−1, *y*+1, *z*; (iii) *x*, *y*, *z*+1; (iv) −*x*+1, −*y*+1, −*z*; (v) *x*, *y*, *z*−1.

## References

[bb1] Arora, V. K. & Knaus, E. E. (1999). *J. Heterocycl. Chem.* **36**, 201–203.

[bb2] Bruker (2009). *APEX2*, *SAINT* and *SADABS*. Bruker AXS Inc., Madison, Wisconsin, USA.

[bb3] Enyedy, I. J., Sakamuri, S., Zaman, W. A., Johnson, K. M. & Wang, S. (2003). *Bioorg. Med. Chem. Lett.* **13**, 513–517. 10.1016/s0960-894x(02)00943-5 12565962

[bb4] Kim, B. Y., Ahn, J. B., Lee, H. W., Kang, S. K., Lee, J. H., Shin, J. S., Ahn, S. K., Hong, C. I. & Yoon, S. S. (2004). *Eur. J. Med. Chem.* **39**, 433–447. 10.1016/j.ejmech.2004.03.001 15110969

[bb5] Knyazhanskii, M. I., Makarova, N. I., Olekhmovich, E. P. & Kharlanov, A. (1996). *Zh. Org. Khim.* **32**, 1097–1103.

[bb6] Kurfürst, A., Lhoták, P., Petrů, M. & Kuthan, J. (1989). *Collect. Czech. Chem. Commun.* **54**, 462–472.

[bb7] Pillai, A. D., Rathod, P. D., Franklin, P. X., Patel, M., Nivsarkar, M., Vasu, K. K., Padh, H. & Sudarsanam, V. (2003). *Biochem. Biophys. Res. Commun* **301**, 183–186. 10.1016/s0006-291x(02)02996-0 12535659

[bb8] Sheldrick, G. M. (2008). *Acta Cryst.* A**64**, 112–122. 10.1107/S0108767307043930 18156677

[bb9] Sheldrick, G. M. (2015). *Acta Cryst.* C**71**, 3–8.

[bb10] Spek, A. L. (2009). *Acta Cryst.* D**65**, 148–155. 10.1107/S090744490804362X PMC2631630 19171970

