# Supplementary figures and images for: Crystal structure of N-[4-amino-5-cyano-6-(methyl­sulfan­yl)pyridin-2-yl]acetamide hemihydrate
Source: Acta Crystallogr E Crystallogr Commun. 2015 Feb 13;71(Pt 3):o171–2. doi: 10.1107/S205698901500256X (PMC4350750; doi:10.1107/S205698901500256X)

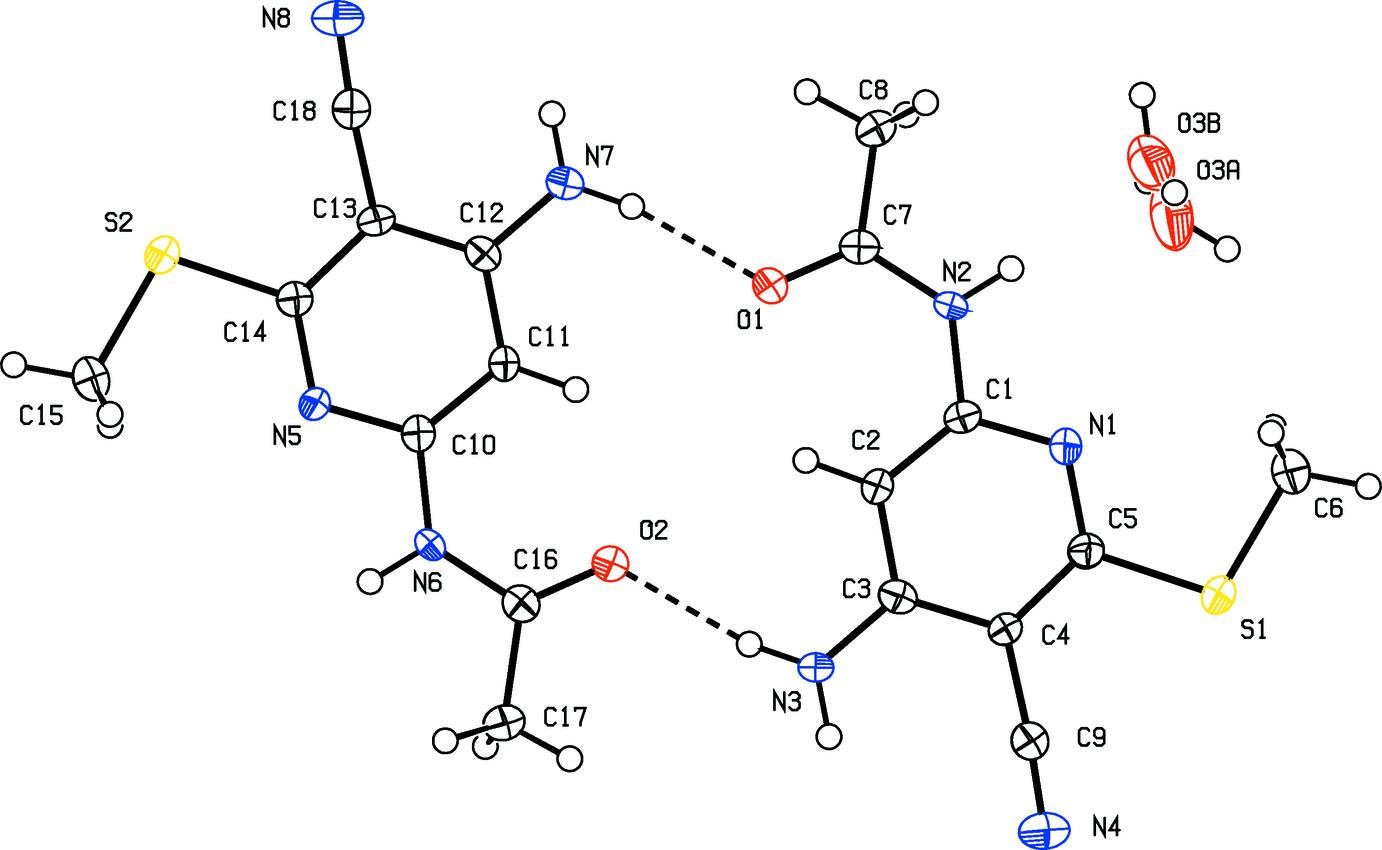

Supplement: Supplementary file 4 [file e-71-0o171-fig1.tif]

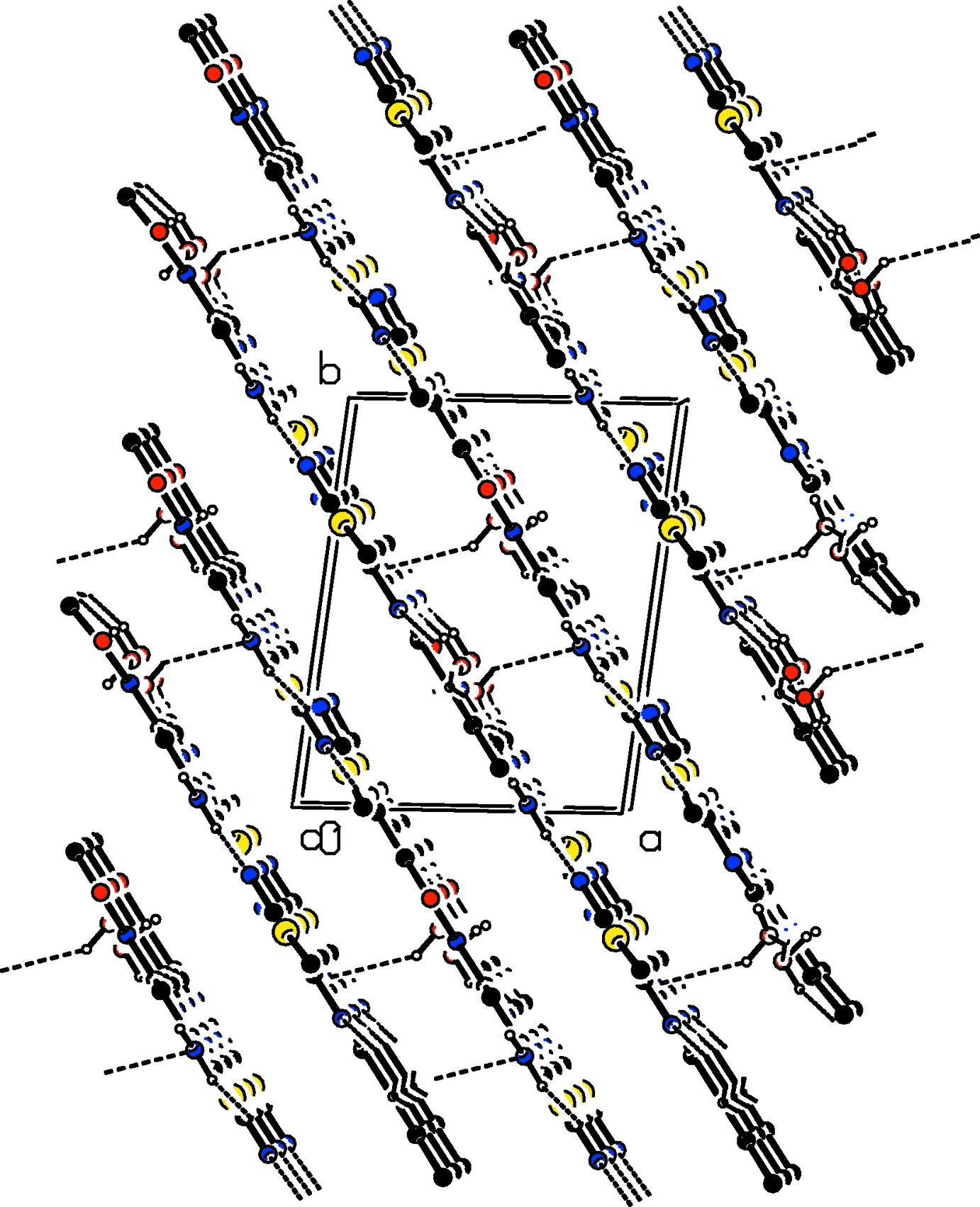

Supplement: Supplementary file 5 [file e-71-0o171-fig2.tif]
